# A Manual of Practical Obstetrics: Arranged so as to Afford a Concise and Accurate Description of the Management of Preternatural Labours; Preceded by an Account of the Mechanism of Natural Labour

**Published:** 1829

**Authors:** 


Art. IX—*A Manual of Practical Obstetrics: arranged so as to afford a concise and accurate description of the Management of Preternatural Labours; preceded by an account of the Mechanism of Natural Labour From the French of Julius Hatin, Doctor of Medicine of the Faculty of Paris, Professor of Obstetrics and of the Diseases of Women and Children, &c. &c. By S. D. Gross, M. D. Philadelphia: Grigg*, 1828. *pp.* 166.

[From a Correspondent]

The author sets out with a judicious definition of preternatural labour, and the object of the practitioner in the management of it, which is to be attained by one of the three means of the *hands*, *blunt* instruments, or *sharp*. The use of the two former, indicated when the maternal parts are well formed, but the dimensions or positions of the fœtal presentations cause the obstacle; thelatter, by implication, although not expressed, solely when the pelvis or other parts of the mother, are deformed. In conclusion of his introductory remarks, he very properly inculcates on the young practitioner, the necessity of a thorough and minute acquaintance with the mechanism of natural labour.

Part 1st. Treats, in a manner not essentially different from that of other approved authors, 1st of the pelvis of the mother, under the divisions of *Anterior*, *Posterior*, and *Lateral regions*. 2nd. Of the Principal parts of the Fœtus, inducing the *motions of its head on the trunk*. 3d. On the Mechanism of natural Labour, in which the Fœtus may present the *head*, *feet*, *knees*, or *breech*. Proceeding, in the order of the presenting parts just mentioned, he gives a circumstantial view of the *four* different modes of presentation of each.

Part 2. Comprehends the management of preternatural labours, the first section of which includes, labours which terminate by mere manual assistance, with *the general causes which induce the practitioner to act;* these are, either on the part of the *mother*, or on the part of the *infant*. It includes also directions respecting *the position of the woman;* and

*for introducing the hand.* The limited character of the work, may account for the paucity of conveniences recommended, preparatory to operation; but the *position of the patient, on the back*, is preferred, in which he coincides with professor Dewees, whose reasons for adopting this position are conclusive.

In the author's *rules for introducing the hand*, after the indispensable *lubrication* of it, and *conical* form, he recommends, that, "in introducing the hand, it should be strongly *pronated*." If, in this position, it gives "least pain," and presents "the least possible volume" relatively, there is certainly great propriety in the direction; it is however questionable; because the *supination* of the hand would seem best adapted to the curvature of the vaginal parts, and the pain occasioned by "bringing it to that state," after the introduction, would be avoided.

The time of, *first*, introducing the hand into the vagina, and, *secondly*, "penetrating the os tincæ," is judiciously distinguished; the practitioner notwithstanding, might have derived benefit from some instruction relative to the *relaxed* state of the *os tincæ* at the time of "penetrating" it, since the effort should never be made, until relaxation has been effected, either by the labour pains, or by venesection, opium, injections, &c. the precaution of fixing the uterus by gentle pressure of its fundus, is very properly recommended.

Presentations of the Feet, are described as principally *four*, which, like those of the head, correspond to the oblique diameters of the pelvis, and are detailed according to Baudeloque. *The characteristics of the feet* are well defined, and *general rules for introducing the hand*, given, in each of the four presentations. With respect to the umbilical cord, which, under some circumstances, for want of necessary relaxation, is required to be *cut*, the author, in imitation of Baudeloque, omits the *ligature*, but it is probably safest, in conformity with the practice of Dewees, to use it. Too much attention cannot be given to the experienced author's circumstantial directions for effecting the delivery of the successive parts of the Fœtus.

Presentation of the Knees, also four, their *characteristic marks described* with sufficient distinctness. The management not being the same in both straits, he therefore distinguishes between the presentations of the *inferior*, and those of the *superior*, giving general rules for the application of the fillet, which for obvious reasons he perfers to the blunt hook. The management in the various presentations of the knees, claims the studious application of the young practitioner, being evidently predicated on much experience.

Next in order are *the presentations of the breech*, in number corresponding with those of the feet, of which minute descriptions are given, in relation both to the *superior* and *inferior* straits; and rules for the application of the *blunt hook* and fillet. In all these the author displays great systematic precision in the management of the labours, under every conceivable circumstance.

Presentations of the Vertex of the Head, are next treated of, in their four principal positions, both at the supeor and inferior straits; also *the characteristic marks of the head*, and the management applicable to its respective presentations. When freeing the superior strait, by pushing the head towards one of the iliac fossæ, he aids the bringing down of the feet, by "*inclining the uterus firmly to the opposite side.*" The *choice of the hand*, in search of the feet, is governed by the side along which it is directed: the hand must invariably correspond by *name* to the *side*, in diagonal presentations. In those direct, either hand may be introduced. The rule laid down by professor Dewees, being more simple, is preferable, namely, the *palm* of the hand employed, should *always* look towards the *face* of the child."

The author proceeds, in order, with Presentations of the Knees, in treating of which, he is, perhaps, too elaborate.— The same evidences, however, of research, and experience, are to be met with in this department of the work. The directions, in relation to the presentation of the *hand*, are brief and judicious.

A recapitulation of the different managements, in each of the presentations, is uniformly given. This first section ends with a synopsis of *Compound Labour*.

Under the SECOND SECTION, of preternatural labours, the author places the INSTRUMENTAL, in which he gives *general rules for the application of the forceps, lever,* and *blunt hook.* The forceps of *Baudeloque* seem preferred, and, doubtless, are calculated entirely to supercede the shorter, or *Haighton's.*

With his characteristic perspicuity, the author describes every variety of presentation of the head, requiring the use of the forceps.

Next, IMPACTION of the head, both in its *occipitofrontal,* and in its *bi-parietal diameter,* with their relative presentations, indications, and management, occupies a considerable portion of this very interesting manual, followed, as usual, by a recapitulation.

SECTION THIRD, embraces *labours which may be terminated by the use of cutting instruments; the particular causes of these kinds of labour,* and the description and use of the *Perforator and Crotchet;* and of the rest, both in relation to the *infant* and the *mother;* are treated of. It is to be regretted, that *Symphysiotomy,* should have found place in this manual, an operation, the advantages of which can never compensate for its performance. Indeed the author's sentiments, if duly weighed, go greatly to the discouragement of it. He observes, "*In summing up the results, it will appear, that symphysiotomy is a serious operation, and that it should be performed only in those instances, where the necessity of the case absolutely demands it*"—which, we are disposed to believe, *never* happen.

To the CAESARIAN OPERATION, although hazardous, no reasonable objection can be made. The author adopts, in conformity with Mauriceau, Platner, Solayres, and others, the *incision upon the linea alba;* and is not wanting in the necessary instructions, in relation to the *instrument; the proper time for performing the operation; the preliminary measures; position of the patient; position of the assistants; operation itself;* and *treatment* afterwards. His summary of the results of this operation, has nothing in it, which ought to deter from, but every thing to recommend, and demand it.

GASTROTOMY is next defined, as the operation sometimes necessary, in consequence of *rupture* of the *uterus,* and *extrau-*

*terine pregnancies*, the *characteristic signs* of which are briefly given; to those of rupture of the uterus, might be added, a *detrusion, wrinkling*, and *coldness* of the *vaginal coat*, which we have, in one instance, observed. The *instruments, place of incision, time of operation*, the *operation* itself, and *treatment afterwards;* are comprehensively noted.

SEC. FOURTH treats of the *artificial delivery of the secundines.* In this section, the author appears to be rather vague and unsatisfactory. Much judgment is sometimes required for the successful management of the placenta. We would take the liberty to refer the student or young practitioner, for necessary information, to professor Dewees's *System of Midwifery.*

On the whole, our profession is much indebted, both to the learned author, and to the translator, of the "*Manual of Practical Obstetrics*," which, we confidently hope, will be in the hands of all, who devote themselves to this important branch of science.

